# Implementation of male-specific motivational interviewing in Malawi: an assessment of intervention fidelity and barriers to scale-up

**DOI:** 10.1136/bmjgh-2024-018269

**Published:** 2026-03-31

**Authors:** Katherine Ničev Holland, Julie Hubbard, Misheck Mphande, Isabella Robson, Khumbo Phiri, Dorina Onoya, Elijah Chikuse, Kathryn Dovel, Augustine T Choko

**Affiliations:** 1 David Geffen School of Medicine, UCLA, Los Angeles, California, USA; 2 Internal Medicine, Beth Israel Deaconess Medical Center, Boston, Massachusetts, USA; 3 Division of Infectious Diseases, UCLA Medical School, Los Angeles, California, USA; 4 Science Department, Partners in Hope Malawi, Lilongwe, Malawi; 5 Health Economics and Epidemiology Research Office, University of the Witwatersrand Johannesburg, Johannesburg, South Africa

**Keywords:** HIV, Public Health

## Abstract

**Introduction:**

Treatment interruption (TI), defined as >28 days late for antiretroviral therapy appointment, is one of the greatest challenges in controlling southern African HIV epidemics. Negative client–provider interactions remain a major reason for TI and a barrier to return to care, especially for men. Motivational interviewing (MI) facilitates client-driven counselling and improves client–provider interactions by facilitating equitable, interactive counselling that helps clients understand and develop solutions for their unique needs. Fidelity of MI counselling in resource-constrained health systems is challenging.

**Methods:**

We developed a male-specific MI curriculum for Malawian male TI clients. Four psychosocial counsellors (PCs, a high-level Malawian counselling cadre) received a 2.5-day curriculum training and job aid to guide MI counselling approaches. PCs implemented the MI curriculum with men >15 years who were actively experiencing TI. Clients were found at home (through tracing) or at the facility (for those who returned to care on their own). MI counselling sessions were recorded, transcribed, translated into English and coded in Atlas.ti V.9. MI quality was assessed using a modified version of the validated MI Treatment Integrity tool. The tool has two measures: (1) counts of key MI behaviours throughout the session (questions, reflections, etc) and (2) overarching scores (using a 5-point scale) that characterise three MI dimensions for an entire counselling session (cultivating change talk, partnership and empathy).

**Results:**

44 MI sessions were recorded and analysed between 1 April 2022 and 1 August 2022. 64% of counselling sessions focused on work and travel as the main reason for TI. 86% of sessions yielded client-driven, tailored solutions for overcoming TI. PCs implemented multiple MI behaviours very well: asking questions, giving information, simple reflections and client affirmation. Few PCs used complex reflection, emphasised autonomy or sought collaboration with clients. Among overarching MI dimensions, PCs scored high in partnership (promoting client-driven discussions) and cultivating change talk (encouraging client-driven language and behaviour change confidence) but scored suboptimal in empathy. Only five sessions had confrontational/negative PC attitudes. Conclusions: PCs implemented MI with fidelity and quality, resulting in tailored, actionable plans for male re-engagement in HIV treatment in Malawi.

**Trial registration numbers:**

NCT05137210 and NCT04858243.

**WHAT IS ALREADY KNOWN ON THIS TOPIC**

- Despite evidence of its efficacy in improving HIV treatment outcomes across the care cascade, person-centred counselling practices, like motivational interviewing (MI), are rarely implemented in low-resource settings due to concerns with respect to fidelity and resource intensity.

**WHAT THIS STUDY ADDS**

- This study provides a systematic analysis of the quality and fidelity of MI provided to men living with HIV in low-resource clinic settings in Malawi by lay-cadre psychosocial counsellors.

**HOW THIS STUDY MIGHT AFFECT RESEARCH, PRACTICE OR POLICY**

- As HIV programmes face ongoing funding constraints, our findings suggest that psychosocial counsellors can deliver high-quality MI with minimal additional resources. This person-centred approach may enhance individuals’ capacity to identify and address their own barriers to care with minimal support, which is particularly important in the current resource-constrained environment.

## Introduction

 Treatment interruption (TI) from HIV care is one of the most pressing challenges to controlling HIV epidemics in eastern and southern Africa.^1^,^3^ In Malawi, TI is defined as being >28 days late for an antiretroviral therapy (ART) refill appointment. In the region, it is estimated that 32%–46% of clients on ART experience TI within the first 5 years on treatment,^4^,^6^ and 30%–40% of clients experience repeat TI.^7 8^ Men experience TI at a 15%–26% higher rate than women^9^ and have worse HIV outcomes like increased morbidity, mortality and viraemia.^10 11^ As such, while countries like Malawi are quickly approaching aggregate UNAIDS 95-95-95 targets, young men aged 15–34 have largely fallen behind.^12^

Resource limitations have been noted to increase rates of provider compassion fatigue and withdrawal,^13^ resulting in poor client–provider interactions that both contribute to male TI and diminish male return to care. ^14^Providers often describe men as being ‘ill-informed’, ‘difficult’, ‘selfish’ and overall ‘bad clients’.^15^ These biases may reflect inequitable client-provider power dynamics and can result in men avoiding HIV services.^14 16^ The hierarchical structure of health systems further entrenches this by positioning providers as the expert on a client’s situation rather than as a collaborator working alongside the client.^13^ The result is predominantly didactic, authoritative counselling that fails to address individualised needs of clients.^13 17^ Given these issues, the WHO has increasingly called for HIV services geared toward person-centred approaches.^16^

Person-centred care (PCC) is an umbrella term for services that centre client respect, client engagement in informed decision-making and support for holistic client needs (physical, psychological and social).^1418^,^20^ Specific PCC techniques, like motivational interviewing (MI), promote responsive counselling and client-driven solutions that may help to address barriers to care. MI is a counselling technique aimed at strengthening a clients’ internal motivation, commitment to behavioural change and development of individualised action plans that promote sustained behaviour change.^21^ MI has been associated with increased ART adherence and decreased incidence of sexual risk taking,^22 23^ and qualitative data suggest client-driven counselling techniques such as MI are deeply desired by male ART clients.^24 25^

Despite its advantages, MI is infrequently used given the extensive resources required that are rarely available in these low-resource settings (eg, increased personnel time, higher level of personnel training and increased monitoring and evaluation). Other challenges to regional implementation include poor fidelity (including poor uptake of ‘the spirit of MI’ and its core behaviours, namely empathy and reflective listening),^26 27^ and poor session attendance.^22^ Furthermore, there is no evidence on MI as a tool for men living with HIV (MLHIV).

We developed a male-specific MI curriculum and training for psychosocial counsellors (PC) to support men experiencing TI in Malawi. We then assessed the quality and fidelity of implementation to determine if PCs could (1) effectively employ MI techniques, (2) counsel with the ‘spirit’ of MI and (3) guide sessions that resulted in actionable next steps for clients to overcome treatment barriers.

## Materials and methods

### Setting

We developed a male-specific MI curriculum for male TI clients and implemented it across eight facilities in Malawi from 1 April 2022 to 31 August 2022. All health facilities were supported by Partners in Hope, a PEPFAR implementing partner that supports HIV service delivery in over 100 facilities serving >200 000 people living with HIV across Malawi. We purposively selected facilities to include large public district hospitals (n=3) and small health centres (n=5), in the central (n=4) and southern (n=4) regions.

### Male-specific MI curriculum

The MI curriculum was composed of two parts. First, an introductory guide was developed to describe the MI approach and help PCs develop the needed skills to implement MI. This included guidance on how to (1) build rapport with clients, including discussing their hopes and goals for the future; (2) support clients to identify their personal barriers to care; (3) explore the potential benefits of treatment adherence and connect these benefits with the clients’ goals; (4) encourage clients’ decision making for change and (5) help clients set their own short-term and long-term goals related to HIV treatment. Our training materials were developed using the REDI Framework and training guide curriculum developed by Engender Health to aid in client contraception choice.^28^ It is one of the few examples of MI-based client-centred care in the region. Second, a male-specific MI job aid was developed to be used during one-time counselling sessions. We used literature on barriers to men’s HIV care^29^,^31^ and formative qualitative research^1532^,^37^ to identify 11 key domains related to barriers to men’s use of HIV treatment. The job aid provided an overview of each topic to sensitise the PC on common challenges to men’s use of HIV treatment and probes to understand the unique barriers, priorities and resources of each individual client. Each domain had MI-related discussion prompts to facilitate client-led discussions and solutions. These were organised by ‘Think’ and ‘Act’ questions. ‘Think’ prompts were used to help the client identify the barrier to care or to reframe the challenge to gain a better understanding of how it could be overcome. ‘Act’ prompts were meant to support the client’s decisions for change and were used to explore actionable ways of overcoming the challenge. A brief description is provided in Appendix A.

### PC training

Four PC, a high-level Malawian counselling cadre, received a 2.5-day curriculum training comprised of (1) sensitisation to the challenges and needs of men as ART clients; (2) education on best practices for male-specific MI, including building rapport, exploring barriers to HIV care, decision-making for overcoming challenges and strategising how to implement them and (3) practice of key MI skills through role play and peer feedback. A key focus throughout the training was the co-creation of actionable next steps for improving clients’ ART adherence. After the training, PC participated in monthly phone-based discussions with their manager for group feedback and discussion about challenges and solutions for implementing the male-specific MI curriculum.

### Implementation

Men who were ≥15 years, living in study facility catchment areas, and not currently in HIV care (defined as never initiated ART or experiencing TI (missed an ART appointment >7 days or defaulted (out of care >28 days)) or having a high viral load (>1000 copies/mL) were offered the male-specific MI curriculum. The latter group was included as persistently high viral load was seen as a likely sign of poor medication adherence despite medications being in hand. The three disparate groups were analysed together because MI seeks to formulate person-specific solutions to individualised problems and should theoretically be adaptable to the myriad reasons for non-adherence. Routine medical charts and health registers were reviewed to identify potentially eligible men. Each PC was responsible for MI implementation at two health facilities. Per standard of care, PC actively traced potentially eligible men to offer male-specific MI counselling. Men were recruited either through this community tracing, or at health facilities if they returned for their ART refill and were flagged as recently having a TI or high viral load. All identified men were screened for eligibility and, if eligible, asked for oral consent, using a script, to record a one-time MI counselling session.

### Measuring Fidelity and Quality in Counselling Sessions: Motivational Interviewing Treatment Integrity tool

We measured the quality of MI components within counselling sessions using a modified version of the Motivational Interviewing Treatment Integrity tool (MITI), a validated scale to assess MI competencies and provide feedback on the fidelity and quality of MI interactions.^38^ We utilised two key measures from the tool: (1) objective counts of key MI behaviours throughout the session and (2) subjective overarching ‘global’ ratings (using a 5-point Likert scale) for domains characterising the ‘spirit’ of MI (see online supplemental Appendix B). Some domains from the tool (including Softening Sustain Talk, Persuade with Permission and Persuade) were not included in our analysis due to the cultural context and the fact that some themes from MITI cannot be measured well via transcripts. Behaviours were classified as ‘simple’ versus ‘complex’ in accordance with whether the behaviour in question is common to traditional counselling (eg, asking questions, giving information, simple reflection, affirmation) versus if the behaviour is more unique to MI-based counselling (eg, complex reflection, seeking collaboration, emphasising autonomy).

In addition to the MITI tool measures, we also assessed PC’s abilities to problem solve and develop next steps to overcome adherence challenges. We rated sessions as ‘effective’ if actionable next steps were created to address the session’s client-identified barriers, or ‘not effective’ if no actionable next steps were created.

### Data analysis

MI counselling sessions were recorded, transcribed and translated into English for analysis. We completed structured memos based on the MITI scale to measure fidelity and quality, including frequency of MI behaviours and ratings of MI domains. Transcripts were reviewed and key data was extracted and entered into the memo, including simple excerpts of text that reflected strong or weak implementation of MITI domains. Transcripts were simultaneously coded in Atlas.ti v.9 using codes for each of the MITI behaviours to aid in the behaviour counts and domains to ensure all content related to themes was captured in their entirety. Client reasons for TI, counts of global rating scores and key MI behaviours per interview were analysed in Excel.

### Patient and public involvement

It was not appropriate or possible to involve patients or the public in the design, or conduct, or reporting, or dissemination plans of our research.

## Results

Overall, 44 counselling sessions were conducted. Counselling session duration ranged from 6 to 60 min, with an average of 27 min (IQR: 18.75). All sessions were completed by male PCs with a minimum of 3 years’ counselling experience prior to the study (mean: 5.65 years’ experience).

### Client type and reason for TI

Client type was recorded for 36/44 counselling sessions. 20 men (45%) had been out of care for >28 days, 15 (34%) had missed an ART appointment by 7 days, 1 (2%) had high viral load and 8 (18%) were unrecorded. Across the 44 counselling sessions, participating men most frequently cited ‘work and travel’ (n=28, 64%) as their main reason for TI. Other reasons for TI included difficulty ‘remembering appointment and medication schedules’ (n=5, 11%) and feelings of ‘loneliness, isolation and lack of social support’ (n=4, 9%).

### Application of key MI behaviours

We measured frequency of the eight key MI behaviours throughout each counselling session. While no ‘target frequency’ exists for any of the MI behaviours, PC most frequently employed simple MI behaviours (eg, giving information, asking questions, simple reflection and affirmation). Advanced behaviours (eg, complex reflection, seeking collaboration and emphasising autonomy) were less frequently utilised. PC overwhelmingly used the seven positive MI behaviours and rarely used the one negative behaviour, confrontation, marked as being antithetical to the ‘spirit of MI.’ Only 11% of counselling sessions (n=5) had one or more instances where the PC demonstrated confrontational behaviour (table 1).

**Table 1:** Key MI behaviour instances

MI behaviour	Mean	SD
Giving information	8.32	7.40
Asking questions	35.45	26.09
Simple reflection	5.18	5.20
Complex reflection	0.80	1.13
Affirmation	4.07	3.56
Seeking collaboration	0.36	0.69
Emphasising autonomy	0.32	0.77
Confrontation (negative behaviour)	0.16	0.53

Mean number of behaviour instances per interview, with variance.

MI, motivational interviewing.

### Most frequently used behaviours

PC relied heavily on the four simple MI behaviours. Giving information and asking questions were the most frequently used. Information was given to resolve knowledge gaps, while asking questions worked to identify these gaps and push the conversation deeper. PC used simple reflection primarily to help clients feel heard and understood. PC echoed clients’ words back to them to indicate that they were paying attention or to give the client opportunity to clarify and reinforce their own thought processes. PC used affirmation to acknowledge a client’s positive attributes and to encourage the positive aspects of a client’s behaviour even when the overarching behavioural theme resulted in negative outcomes, like TI (table 2).

**Table 2:** Qualitative examples of frequently used MI behaviours in action

MI behaviour	Utility	Quote
Giving information	Resolve knowledge gaps	**PC:** *I also want to remind you to take your health passport with you when you travel long distances so when you run out of drugs, you can get an emergency supply from the nearby facility there*.
Asking questions	Identify knowledge	**PC:** *Do you also have other things that you would like us to discuss?* **Client:** I was told that if you adhere to treatment, you reduce risk of transmitting the disease to your wife, if you are not sleeping around. Is it true?
	Deepen the conversation	**Client:** *I was sick*. **PC:** *Can you explain?* **Client:** *The illness left me unable to walk; I had to be carried—I started swelling and had wounds which were not healing. When I realized my body was deteriorating, I restarted the medication*
Simple reflection	Make clients feel heard and understood	**PC:** *Thank you for your explanation. Just to see if I have understood you well, you have said in the morning you take one pill per day and in the evening, Bactrim*.
	Clarify discussion points	**PC:** *Can you clarify the point where you have referred to yourself as being ‘blinded by the devil?’*
		**PC:** *If I have got it correctly, you have taken two children from your first marriage, the other children died. But you don’t have a child with the current wife?*
	Recapitulate core client points	**PC:** *Let me just recap what you have said. I appreciate that you have explained you are aware of the importance of taking ARVs. You said that if one doesn’t take ARVs, the body is never healthy, and you don’t look good*.
Affirmation	Acknowledge positive client attributes	**PC:** *You can even be an example to others that are struggling with treatment for you claim that you don’t hide it*.
		**PC:** *Thank you. Let me thank you very much because of how you are making your explanations. It shows that you followed the counseling from the beginning meticulously. Because if you had not listened to the counseling or if you didn’t know, you wouldn’t have been able to speak like you are doing today*.
		**PC:** *You have done a very important thing. It is indeed important to have something that can be reminding you so that you don’t miss your dates*.
	Acknowledge positive aspects of client behaviour despite overarching negative behavioural theme	**PC:** *Let me also appreciate that when you heard the message you didn’t hesitate, there was good communication and you also followed through with the promise that you made saying you will come on a certain day, and you have come today. For that, we thank you. You explained very well that there was a mistake because you went to (another clinic, not your home clinic)*.
		**PC:** *First of all, we should appreciate you for coming to the hospital immediately after you arrived yesterday because you were asked to be here. You were tired with the trip, but you still made sure that you should respect our call*.

Summary of most used MI behaviours, with example quotes.

MI, motivational interviewing.

### Least frequently used behaviours

PC rarely used advanced MI behaviours. When used, complex reflections allowed PC to distill a client’s thought into key points, pushing the conversation forward by both extracting and emphasising salient emotions and themes that arose throughout the client’s words. Even rarer was the use of seeking collaboration. However, when used, it emphasised power sharing and allowed the client to drive the conversation in a direction most suitable for their personal needs. Emphasising autonomy was the least used positive MI behaviour among PC. Despite this, its implementation was critical to fostering client buy-in by dispelling notions of being forced or shamed into regular medication adherence.

While infrequent, confrontation (negative MI behaviour) did occur. PC reverted to confrontational language when they felt their clients were not being honest and when they felt they needed to correct behaviours they viewed as negative (table 3).

**Table 3:** Qualitative examples of infrequently used MI behaviours in action

MI behaviour	Utility	Quote
Complex reflection	Emphasise and push conversation forward	**PC:** *It seems that the fears are there, that is why you come here [to this hospital instead of a closer hospital*]. *However, this suggests to me that there might be others, as well, from where you come from who can also come here to seek medical attention (but don’t)*.
Seeking collaboration	Emphasise power sharing	**PC:** *I am someone who is readily available to assist you. Do you have any comment or concern that we should discuss? Something that can be helping you in your daily life?*
		**Client:** *I take my medication at 7am but my worry is on what will happen if I am late with an hour, what will be the result and how can I go about it?* **PC:** *I will come in but before that, what do you think?* **Client:** *There are other medications which have a period. If you are within the specified hours, you can take them. With other medications, it is rumored that you can take two tablets instead of one but let me learn from you on this one. I guess this is why we are here, as men, to discuss these issues. I have never been counselled regarding this and I have never asked before. I guess this is my chance*
		**PC:** *Going back a bit, you asked me about the sort of care that you will be getting with respect to the treatment, considering also that you have epilepsy. Would you like us to discuss those issues now?* **Client:** *It is possible because I want to understand and become aware*.
Emphasising autonomy	Foster buy-in	**PC:** *We are all men and when discussing, we understand each other. You also have the right to say what you think. Final decision comes from you. We don’t have a problem with that*.
		**PC:** *As you are aware, we are not as strict as the police here at the hospital. We recommend what you should do in the treatment, and we rely on you to be a responsible person and adhere. However, the choice is yours*.
Confrontation	Entrench power hierarchies	**PC:** *There should be something that made you afraid. You need to be open with me*.
		**PC:** *What should be done, then? Because I feel like you are imposing your responsibility on her, which is not okay. You will be the one suffering for not adhering. You should claim ownership and let the wife be there only to assist*.

Summary of least used MI behaviours, with example quotes.

MI, motivational interviewing.

### Creating actionable next steps

Across the 44 counselling sessions, 91% of counselling sessions resulted in actionable next steps for at least one barrier to care and 86% developed solutions for all identified barriers (see online supplemental Appendix C examples). With the support of PC, men developed plans for overcoming barriers to care including disclosure to close male friends, posting ARV reminders on their bedroom walls, and having a plan in place for unanticipated travel, among others.

Four out of the 44 sessions did not yield actionable steps; the sessions identified as failing to yield next steps were all focused on ‘work and travel’ as the primary barrier to care. Of the four instances where no next steps were given for the topic of ‘work and travel’, two involved the client not having adequate transport to attend the facility and two involved one-off instances of illness while travelling which prevented return to the facility. Counselling discussions suggested particular difficulty overcoming time and cost associations with work and travel.

### Quality of MI by global ratings

Global ratings for elements that represented the ‘spirit of MI’—cultivating change talk, partnership and empathy—were graded on a 1 (lowest) to 5 (highest) scale for each counselling session (table 4).

**Table 4:** Central tendency and spread of global rating scores per interview

Global rating	Mean score	SD	Median score	Mode score
Cultivating change talk	3.16	1.72	3	5
Partnership	3.80	1.44	4	5
Empathy	2.75	1.28	3	3

Mean, median and mode global rating score per interview, with variance.

### Partnership

PC generally scored best in partnership, or their ability to convey an understanding that expertise and wisdom about change resides mostly within the client, with a mean score of 3.8. PC displayed partnership through statements that highlighted wisdom residing in the client themselves and by using terminology that offered the client an equal position in the discussion. When PC used language to convey partnership, it elicited higher-level engagement from the client; rather than simply listening, the client began to actively participate and offer their critical thoughts on the subject at hand. This helped build rapport and the client’s sense of agency in crafting a plan toward treatment re-initiation. Most commonly, partnership came in the form of questions that evoked solutions from the client, but it occasionally came in the form of explicit statements calling for equality.

Despite scoring well in partnership, several exchanges between clients and PC signalled that clients were resistant to, or initially did not accept, the PC’s bid for partnership. One such example is seen below where the client appears to be either expecting or asking for a more traditional, didactic interaction with the PC:

PC: The medication is not compatible with you. Okay, so do you know how we solve this problem?

Client: You tell me.

### Cultivating change talk

PC also achieved good scores for cultivating change talk, marked by the mentor actively encouraging the client’s own language in favour of the change goal, with a mean score of 3.16.

Cultivating change talk occurred through extended back and forth dialogue between the PC and client whereby the PC would help identify a cause of TI and explore the barrier with broad, open-ended and evocative questions that required the client to consider the issue at hand from multiple angles. The result was often that the client would come to a conclusion that aligns with traditional PC recommendations but on their own accord.

### Empathy

PC received the lowest scores for empathy, with an average mean score of 2.75. When employed, PC used empathic language to demonstrate that they saw the client as a human and understood that perfection is not always a reasonable goal. They emphasised that one must give themself grace while on treatment— the client should not wait to start treatment until they can adhere perfectly; everyone forgets at times and persevering is what matters. PC also expressed empathy by apologising on behalf of the health system for negative experiences the client endured in the past even when the PC was not directly responsible for the negative interaction. Mostly commonly, empathy was expressed in the form of ‘as men’ statements which sought to express to the client that the PC understood, as a fellow man, that there are many conflicting responsibilities that can make adhering to treatment particularly difficult (table 5).

**Table 5:** Qualitative examples of global rating dimensions

Global rating	Utility	Quote
Partnership	Evoke client-driven solutions	**PC:** *You said you started taking ARVs a few years ago. In your own opinion, how would ARV adherence help with your other responsibilities in life?* **Client:** *It would give me longer life and allow me to both care for my children and carry out the responsibilities I was elected for in my village*.
		**PC:** *What have you learned from this situation? How can we solve this problem [of ART adherence]?* **Client:** *Firstly, I have indeed missed the full month. In past times, we used to leave our phone numbers here and they would either call or visit us as a reminder, often offering ART adherence counseling, as well*.
	Explicitly call for equality	**PC:** *You call me (PC’s first name). Don’t call me boss but take me as a fellow man who wants to chat and discuss two or three things with you. You will learn something from me, and I will learn something from you, too*.
		**PC:** *What should we do so that whenever such trips happen you shouldn’t compromise your treatment? I just want us to understand what prevented you from coming for refill so that together we can sort out and prevent it from happening again next time*.
Cultivating change talk	Facilitate client-driven language in favour of change goal	**PC:** *Based on what you shared, you missed your appointment because you were sharing medications with your wife, correct?* **Client:** *Yes*. **PC:** *When your wife is given a 3-month refill and you share with her, what happens?* **Client:** *From my experience, it causes a huge problem for us and the psychosocial counselors because we end up missing appointments, and it has consequences*. **PC:** *Do you think sharing medication is a good decision?* **Client:** *It is not a good because when we are given appointments, the psychosocial counselors calculate how much medication we need for that period. When we share medicine, one person will not have enough*. **PC:** *What should be done then? As you said, sharing medicine with your wife disturbs both of your schedules*. **Client:** *I should talk with my wife and have her return to the facility since her dates and medications no longer align*.
Empathy	Emphasise grace on treatment	**PC:** *It is bound to happen because people are not computers. [They] happen to forget*.
		**PC:** *We are all humans, and by that, we do forget things. That is normal*.

Summary of global ratings, with example quotes.

## Discussion

We assessed quality and fidelity of male-specific MI curriculum implementation by PC in routine HIV care settings in Malawi and found that PC were able to achieve both quality and fidelity in certain areas. Following a brief training, PC could (1) effectively use most MI behaviours with rare instances of confrontation, (2) demonstrate successful application of MI domains and (3) guide counselling sessions that resulted in actionable next steps for clients to overcome TI. However, PC demonstrated infrequent usage of advanced MI behaviours and did not display high rates of empathy with clients, suggesting that additional training or altered curricula may be needed to promote advanced MI behaviours and global skills.

When implementing male-specific MI, PC in our study overwhelmingly relied on simple behaviours (asking questions, giving information, simple reflection and affirmation). Despite their simplicity, these simple MI behaviours seemed to engage male clients by clearing knowledge gaps, making clients feel seen and heard, and affirming clients. Advanced MI behaviours (complex reflection, seeking collaboration and emphasising autonomy) were used less frequently but served to deepen conversations, centre counselling on the unique needs of the client and reaffirm that enduring solutions reside within the client, not the PC. Infrequent usage of advanced MI behaviours may reflect ingrained client–provider power dynamics and hierarchies that position providers as experts meant to direct, rather than collaborate, with a client.^13^ Infrequent usage of complex behaviours may also reflect the need for additional training to advance the nuanced counselling skills necessary for deeper MI implementation. PCs’ ability to engage in effective MI despite minimal use of complex MI behaviours suggests that simple, pared down MI behaviours may be sufficient to instil the ‘spirit of MI’ into a counselling session in contexts such as Malawi. While infrequent, rare confrontational exchanges worked to entrench typical counselling hierarchies and fortify the unequal power dynamics that MI seeks to dismantle, thus creating a more hostile environment not conducive to open dialogue.

Despite most interviews resulting in actionable next steps and being rated as ‘effective’, there was wide fluctuation in behaviour frequency across interviews, suggesting that there is no ‘right number’ of behaviour frequencies required to enact high-quality MI. Frequencies of usage should be expected to be variable, fitting with the spirit of MI, which seeks to provide an individualised approach rather than a ‘one-size-fits-all’ approach. Effective counselling sessions also varied considerably in length, further supporting that there is no one-size-fits-all approach to quality, effective MI.

Forming actionable next step plans was critical to enacting effective MI. 91% of counselling sessions resulted in actionable next steps for at least one barrier to care and 86% developed solutions for all identified barriers. With the support of PC, men developed plans for overcoming barriers to care including disclosure to close male friends, posting ARV reminders on their bedroom walls, and having a plan in place for unanticipated travel, among others. While rare, some sessions failed to develop plans for overcoming; this overwhelmingly occurred when the reason for TI was ‘work and travel’, which was also the main identified reason for TI among study participants. Counselling sessions suggested that time and cost associations with work and travel may be particularly challenging to address due to their logistical nature, particularly in low-resource settings. Other literature has similarly found that many clients experience HIV TI in part due to insufficient affordable transport solutions and conflicting financial demands which are not easily resolved given current infrastructure and socioeconomic realities.^39^,^41^ Additional novel structural interventions at the health facility level, like home ARV delivery or multimonth dispensing, may be needed to overcome these barriers, if feasible in the local context.

PCs were largely proficient in two of the three core elements that represent the ‘spirit of MI’, partnership and cultivating change talk, after only 2.5 days of training. Despite success in those domains, PC scored worse in empathy. This may reflect several challenges including, but not limited to: (1) cultural practice of expressing empathy in less explicit or non-western manners^42^, (2) PC practice of expressing empathy through body language rather than words, thus not flagging in transcripts or (3) insufficient training in engaging MLHIV with empathetic statements.

The overwhelming proportion of study counselling sessions developed actionable next steps and were ultimately rated as ‘effective’, suggesting that brief trainings focused on developing MI behaviours and low-cost job aids which remind PC of basic MI principles may be effective tools to aid basic MI implementation in routine settings. As HIV programming transitions back to ministries of health from PEPFAR,^43^,^45^ these results bode well for continued implementation; it is possible to provide high-quality, skilled MI counselling with little resources and time with minimal upfront investment.

## Conclusions

Our findings are critical for understanding the feasibility of providing high-quality MI counselling in resource-limited settings and will only become more relevant as funding for HIV programming across the continent becomes more constrained. Our results suggest that PC can implement high-quality MI with fidelity in a low-resource setting with brief training. While largely successful, additional training or altered curricula may help promote the lesser-seen behaviours and global skills, like complex reflection or empathy. Further, ‘work and travel’ remains a chief barrier to treatment among MLHIV and a particularly challenging barrier to care for PC and clients alike to overcome. Future work should focus on taking male-specific MI counselling to scale in the standard of care through continually simplified trainings, curricula and monitoring and evaluation strategies. Additional areas of future focus include exploring challenges to PC engagement with advanced MI skills, like complex reflection and empathy.

## Supplementary material

10.1136/bmjgh-2024-018269 online supplemental file 1

10.1136/bmjgh-2024-018269 online supplemental appendix 1

## Data Availability

Data are available in a public, open access repository.
