# Supplementary material for: Implementation of male-specific motivational interviewing in Malawi: an assessment of intervention fidelity and barriers to scale-up
Source: BMJ Glob Health. 2026 Mar 31;11(3):e018269. doi: 10.1136/bmjgh-2024-018269 (PMC13052563; doi:10.1136/bmjgh-2024-018269)
Supplement: online supplemental file 1 [file bmjgh-11-3-s001.docx]

**Reflexivity Statement**

1. **How does this study address local research and policy priorities?**

This study aligns with local (health systems) and national (Malawian Ministry of Health) goals / priorities of developing and identifying interventions capable of decreasing the nation-wide burden of HIV and improving outcomes across the care cascade. It furthers aligns with the goals of the Ministry of Health as it looks to provide insight into the efficacy of person-centered care in low-resource settings, providing the groundwork for future studies on ultimate cost-effectiveness and scalability of the intervention in such regions.

1. **How were local researchers involved in study design?**

Local Malawian researchers (MM, EC) collaboratively worked with research partners in the USA (JH, KD) and Great Britain (IR) to develop the male-specific counseling curriculum intervention as well as the study implementation protocol.

1. **How has funding been used to support the local research team?**

The funding of this study has supported three local Malawian researchers (MM, KP, EC) and provided partial support for senior researchers’ time (AC) at the partner organization in Malawi, Partners in Hope.

1. **How are research staff who conducted data collection acknowledged?**

The primary data collectors involved in the study are acknowledged as co-authors (JH, MM, IR) and were active members of the study team from inception to dissemination.

1. **Do all members of the research partnership have access to study data?**

All members of the partnership have access to the data.

1. **How was data used to develop analytical skills within the partnership?**

Data was analyzed by two early career researchers (KNH and JH) from the USA with later input and commentary from early and seasoned Malawian researchers upon review of manuscript drafts (MM, KP, EC, DO, AC) allowing bilateral growth in data analytics.

1. **How have research partners collaborated in interpreting study data?**

The study team met weekly since the inception of the study to jointly plan study design, data collection methods, analysis, interpretation, and dissemination of results. All members of the partnership were involved in review of the final study data and findings.

1. **How were research partners supported to develop writing skills?**

The research partners worked collaboratively on numerous national and international oral and written presentations throughout the entirety of the study. Group meetings and discussions allowed opportunities for support and growth in areas inclusive of writing and communication skills across modalities.

1. **How will research products be shared to address local needs?**

The results of the study have and continue to be shared across numerous modalities inclusive of academic papers, presentations, and conferences at the local, national, and international level. Findings were also specifically disseminated with the Malawian Ministry of Health.

1. **How is the leadership, contribution and ownership of this work by LMIC researchers recognized within the authorship?**

Authors MM, KP, EC, AC are all nationals of Malawi and author DO is a national of South Africa. MM and EC were involved in development of the intervention while MM, KP, DO, EC, and AC were all involved in editing and approval of the final manuscript.

1. **How have early career researchers across the partnership been included within the authorship team?**

Early career researchers from both Malawi (MM, EC) and the USA (KNH, JH) have been involved in the authorship team.

1. **How has gender balance been addressed within the authorship?**

Six main authors are women (KNH, JH, IR, KP, DO, KD) and three main authors are men (MM, EC, AC).

1. **How has the project contributed to training of LMIC researchers?**

The study intervention development was collaboratively crafted between early career researchers in Malawi under the guidance of advanced career researchers in the USA. Implementation of the study intervention and data collection was also completed by early career researchers in Malawi alongside early career researchers in the USA under the guidance of advanced career researchers in both Malawi and the USA.

1. **How has the project contributed to improvements in local infrastructure?**

This study provided the grounding for later projects aimed at scaling up person-centered counseling strategies within Malawi. This study also continued to strengthen standing long-term research partnerships between study partners which have continued beyond the study in question.

1. **What safeguarding procedures were used to protect local study participants and researchers?**

The study was conducted in accordance with ethics approval from the UCLA and NHSRC Malawi IRB review board under the IDEaL trial (Reference Number: NCT05137210). Participants underwent informed consent prior to inclusion in the study, and all participant data was collected confidentially prior to being de-identified in the results. Weekly meetings with all involved researchers allowed for open discussion and reflection over the course of the study and promoted ongoing strengthening of the existing relationships while continuing to foster trust and open communication.
