# Supplementary material for: Implementation of male-specific motivational interviewing in Malawi: an assessment of intervention fidelity and barriers to scale-up
Source: BMJ Glob Health. 2026 Mar 31;11(3):e018269. doi: 10.1136/bmjgh-2024-018269 (PMC13052563; doi:10.1136/bmjgh-2024-018269)
Supplement: online supplemental appendix 1 [file bmjgh-11-3-s002.docx]

**Appendix A: Topics & Descriptions in the Male Specific MI Curriculum**

| **Domain** | **“Think” Prompt** | **“Act” Prompt** |
| --- | --- | --- |
| **Stigma & Discrimination** | Remember, HIV is a virus that affects people worldwide. There are others who have the same fears as you. You are not alone. | Do you know anyone else who is living with HIV? Let’s make a plan for you to ask to talk to them about their experience with HIV stigma and how they manage it. |
| **Status Disclosure** | Do you know anyone who has disclosed successfully? If so, how did they do it? How did they overcome their fears? If they disclosed to you, how did it make you feel? | Think about someone you want to disclose your HIV positive status to. Now let’s role play this scenario together. |
| **Disbelief & Challenges with Status Acceptance** | There are many people out there who choose not to test. You are being a responsible person who is choosing to know your status. | If you are struggling to accept your status, it may be helpful to get re-tested. This is always an option – would you like to make a plan ask the provider to re-test? |
| **Poor Knowledge of ART** | Think about things about ART that you want to understand. Do you want more information about how to protect a sexual partner? Or are you curious about how you can have children now that you are HIV positive? | Let’s go through your list of questions together and I will answer any of your questions I am able to answer. If I don’t have the answer, we can go to the provider together to ask so we both are able to learn. |
| **Side Effects** | Many medications, such as medication for bacterial infections, Malaria, TB, cold and flu, have a risk of some side effects as our bodies adjust. IARVs can be similar. How have you overcome other side effects in the past? | Some side effects can be overcome with other medications that your provider can provide (exp: anti-nausea medication). Let’s make a plan to ask your provider what medications are available to help with side effects. |
| **Loneliness, Isolation, & Lack of Social Support** | It can be good to realize you are not alone, that these feelings are human regardless of HIV status. Do you know anyone else, HIV positive or not, who has experienced loneliness or a lack of support? | This week, let’s make a plan for you to talk to one of your trusted friends about how they can help you feel supported. |
| **Taking ART when Feeling Healthy** | We often think that ART is only for sick people. But this is not true! ART is for anyone who is HIV positive, regardless of whether they are sick or well. | Would you like to start ART today? |
| **Lifelong Medication** | The grasshopper can travel long distances even with small jumps. Every day is a small step. Being on treatment for life may feel long, but taken bit by bit, it is manageable. | Taking medication everyday can be less burdensome when it’s integrated into your daily habits. What time is best for you to take your medication? Let’s link that time to a habit that you do every day (brushing your teeth in the morning, taking tea, etc). |
| **HIV, Work, & Travel** | We as men need to juggle many responsibilities at once. We can manage work commitments while still managing responsibilities like going to church, being a leader in the community, taking care of our families. Can you describe the commitments you have and how you keep these commitments and work at the moment? | What do you do if you need to take your medication at work but don’t want others to see it? Wrap the pills that you need in a small cloth pouch or plastic zip bag and keep the pills in your pocket or a safe location (i.e. a backpack) while you are away from home. |
| **Lifestyle (alcohol, etc)** | We know we can’t change everything about our behaviors at once. That’s okay. But we can make small changes every day to ensure we are living the as healthy as we can. | Think about small changes that you can make to your lifestyle to take a step towards better health (i.e. one less drink/pack of cigarettes per week, making a conscious decision to be prepared with condoms before going out, etc). What’s your change? |
| **HIV clinics** | Most health programs focus on children or mothers. But YOUR health as a man is equally important because it is the main vehicle towards reaching your goals in life and keeping your family well. Think about yourself. Do you prioritise your health? How do you do this? | Next time you’re at the clinic for routine health for yourself or for someone else, consider starting ART. You are already there – you’ve taken the time, effort, and spent the transport money to attend. You can make the trip work double for you by deciding to start during a visit you are already at the clinic. |

**Appendix B: Domains Included in the MITI Scale & Definitions**

| **Behavior Count** | **Definition** | **Example** |
| --- | --- | --- |
| **Giving information** | Mentor gives information, educates, provides feedback, or expresses a professional opinion without persuading, advising, or warning. Tone is usually neutral. | “It is recommended that you take your medication at the same time every day.” |
| **Asking Questions** | Any question that is asked. Includes fact-finding, close-ended, and open-ended questions that cannot be answered with yes or no and are phrased in a way that require further explanation from the client. | “What challenges are you facing when it comes to taking your medications?” |
| **Simple Reflection** | Instances where the mentor reflective listening statements in response to client statements. Simple reflections typically convey understanding or facilitate client–mentor exchanges. These reflections add little or no meaning (or emphasis) to what clients have said. | “You seem discouraged.” “Things seem very stressful for you.” |
| **Complex Reflection** | Instances where the mentor uses reflective listening statements in response to client statements. Complex reflections typically add substantial meaning or emphasis to what the client has said. These reflections serve the purpose of conveying a deeper or more complex picture of what the client has said. | “It seems like it’s really hard for you to take medication every day.” |
| **Affirmation** | Instances that accentuate something positive about the client. To be considered an Affirm, the instance must be about client’s strengths, efforts, intentions, or worth. | “It sounds like you have worked really hard to stay adherent when traveling.” |
| **Seeking Collaboration** | Instances where the mentor explicitly attempts to share power or acknowledge the expertise of the client. The key here is that the mentor genuinely seeks consensus with the client regarding tasks, goals, or directions of the session. | “Since you have not disclosed your status, would it be alright if we spent some time discussing your relationship with your family/those close to you?” |
| **Emphasizing Autonomy** | Instances where the mentor clearly focuses the responsibility with the client for decisions about and actions pertaining to change. They highlight clients’ sense of control, freedom of choice, personal autonomy, or ability or obligation to decide about their attitudes and actions. | “You are the only one who can decide which direction your life will go” “At the end of the day, it’s up to you to take your medication every day.” |
| **Confrontation (Negative Interaction)** | Mentor directly and unambiguously disagrees, argues, corrects, shames, blames, criticizes, labels, warms, moralizes, ridicules, or questions the client’s honesty. Such interactions will have the quality of uneven power sharing, accompanied by disapproval or negativity. | “You said you drink multiple times during the week. This is way too much alcohol. That is not good.” |

**Appendix C: Next Step Action Plans**

| **Reason for Treatment Interruption** | **Number of Clients with a Particular Reason for Interruption** | **Number of Instances with “No Next Steps” Established** | **Example of an Established “Next Step” for Mitigating Future Treatment Interruption** |
| --- | --- | --- | --- |
| Fear of Disclosure | 1 | 0 | Client is an elder and a chief and this status puts him in a precarious position when it comes to waiting in a public line for ARVs 🡪 Clinician recommends using a guardian to obtain medications. |
| Side Effects | 2 | 0 | Client states they previously had bad reactions to ARVs 🡪 Clinician informs client that there are new medications with lower side effect profiles. Clinician also informs client that, in the event of a medical issue like medication side effects, client should call the hospital so they can be seen by a doctor. |
| Loneliness, Isolation, & Lack of Social Support | 4 | 1 | Client’s second wife recently passed away. He is not on good terms with his first wife. Further, his biological children are far from him and his stepson in an Evangelical Christian who does not believe in hospitals and thus won’t go to them. Client feels lonely in his diagnosis 🡪 Clinician suggests that client reach out and disclose to a male friend or male relative who may be able to act as a guardian. Client thinks this is feasible and plans to do so. |
| “But I Feel Healthy” | 1 | 0 | Client reaffirms that he feels healthy and is not in need of medications 🡪 Clinician uses a metaphor about a house that looks nice on the outside but is being destroyed by termites on the inside to express that even if you look or feel healthy now, your body is sick. Client says they understand now, and they will start taking medications so that “things should go smoothly.” |
| HIV & Other Responsibilities: Work & Travel | 28 | 4 | Client missed their appointment because they had to return to their childhood home to help their sick mother cultivate the maize 🡪 Clinician reminds client that, in the future, when going away from their current home they can use several methods to obtain ARVs: (1) take a health passport and collect ARVs at a hospital near where they are staying, (2) use a guardian to gather medications, or (3) upon gaining trust of a provider, consider asking for multi-month medication dispensing |
| HIV Clinics | 3 | 1 | Client states that clinic lines are too long, and they don’t have time to wait 🡪 Clinician informs client that clinic is typically much quieter after 12pm and simply arriving to clinic later in the day could resolve the client’s concerns.  Client expresses immense fear of the HIV clinics and explains that his friend was once screamed at for not properly adhering to his ARVs 🡪 Clinician apologizes for the friend’s experience and lets the client know that each clinic has a suggestion box available if someone has a poor experience. Clinician then walks the client through avenues the client can take to express their feelings to a clinic manager. |
| Sharing Medications | 3 | 0 | Client did not come to clinic because his wife also have HIV and he used some of her medications rather than picking up a refill 🡪 Clinician suggests having his wife come to clinic with her passport in order to receive additional ARVs to make up for those shared, client agrees to set up reminder calls with his clinic so he is not in the scenario of running out of ARVs and needing to use someone else’s, and client decides to use a calendar to record upcoming appointment dates. |
| Sickness Related Issues | 2 | 0 | Client missed their appointment because they were feeling sick and came to clinic once they felt better 🡪 Clinician states that, in this scenario, the client did the right thing to not send a guardian because the client also needed to get a blood draw. Clinician affirms that client should do the same thing next time (simply come to clinic as soon as he feels ok again) so long as they have enough medication around. |
| Trouble Remembering Appointments & Medication Schedule | 5 | 1 | Client states they are having difficulty remembering to take their medications 🡪 Clinician and client brainstorm ideas for remembering and client decides to post a timetable on their wall to remind them. |
| Conversations about Lab Tests | 1 | 0 | Client did not understand what their lab tests meant and thus did not come to clinic 🡪 Clinician walks the client through how to read and interpret the HIV lab tests and explains that clients should ask someone at the facility, upon receipt of the results, to aid in test interpretation if there is any confusion. |
| Issues Accessing HIV-Related Services | 2 | 1 | Client had issues navigating HIV services 🡪 Clinician offers to escort the client in real time to pick up the client’s medications so that the client will feel comfortable going forward. |
| Familial / Personal Issues | 1 | 0 | Client explains that, upon initial receipt of his diagnosis, he was not mentally or emotionally prepared to undergo treatment. He is currently experiencing marital strife, though, and for fear of the fate of his children, he wants to start treatment 🡪 Clinician reexplains to the client how to take his medications properly and counsels on what to do if the client must leave town. |
| Transportation | 2 | 0 | Client explains that they did not come to their recent appointment because they ran out of money 🡪 Clinician and client brainstorm and decide that the client should set aside some money each month which can be used for transport. When their appointment is nearing and they find that the money is still available, they should come to the hospital while the money is available even if it means coming earlier than the set appointment date. |
